# The Role of Myxoma Virus Immune Modulators and Host Range Factors in Pathogenesis and Species Leaping

**DOI:** 10.3390/v17081145

**Published:** 2025-08-21

**Authors:** Junior A. Enow, Ana M. Lopes, Joana Abrantes, Pedro J. Esteves, Masmudur M. Rahman

**Affiliations:** 1 Biodesign Center for Personalized Diagnostics, Biodesign Institute, Arizona State University, Tempe, AZ 85287, USA; jenow@asu.edu; 2 School of Life Sciences, Arizona State University, Tempe, AZ 85281, USA; 3 UMIB-Unit for Multidisciplinary Research in Biomedicine, ICBAS-School of Medicine and Biomedical Sciences, University of Porto, 4050-313 Porto, Portugal; analopes.research@gmail.com; 4 ITR, Laboratory for Integrative and Translational Research in Population Health, 4050-600 Porto, Portugal; 5 CIBIO, Centro de Investigação em Biodiversidade e Recursos Genéticos, InBIO Laboratório Associado, Campus de Vairão, Universidade do Porto, 4485-661 Vairão, Portugal; jabrantes@cibio.up.pt (J.A.); pjesteves@cibio.up.pt (P.J.E.); 6 BIOPOLIS Program in Genomics, Biodiversity and Land Planning, CIBIO, Campus de Vairão, Universidade do Porto, 4485-661 Vairão, Portugal; 7 Departamento de Biologia, Faculdade de Ciências, Universidade do Porto, 4099-002 Porto, Portugal; 8 CITS—Center of Investigation in Health Technologies, CESPU, 4585-116 Gandra, Portugal

**Keywords:** poxvirus, leporipoxvirus, myxoma virus, immune modulators, host range, species leaping

## Abstract

Myxoma virus (MYXV) is a leporipoxvirus that causes lethal disease in Leporids. Hares and rabbits belong to the Leporidae family and are believed to have had a common ancestor 12 million years ago. After seventy years of contact with European hares without causing mortalities or disease manifestation, a recombinant MYXV infected Iberian hares (*Lepus granatensis*) causing high mortalities. Like all poxviruses, MYXV encodes a wealth of immune modulators required for successful virulence that also mediate host species jumping, for example, into hares. Here, we summarize the data of known MYXV immune modulators, their cellular functions, and their effects on European rabbits. Additionally, we suggest that the critical restrictions MYXV would encounter in colonizing a potentially new host species stem from their interactions with the host’s innate immune environment. Lastly, we synthesize our understanding of some poxvirus genome architectural features that might have facilitated the host species jump of MYXV into hares from rabbits.

## 1. Introduction

**Figure 1:**
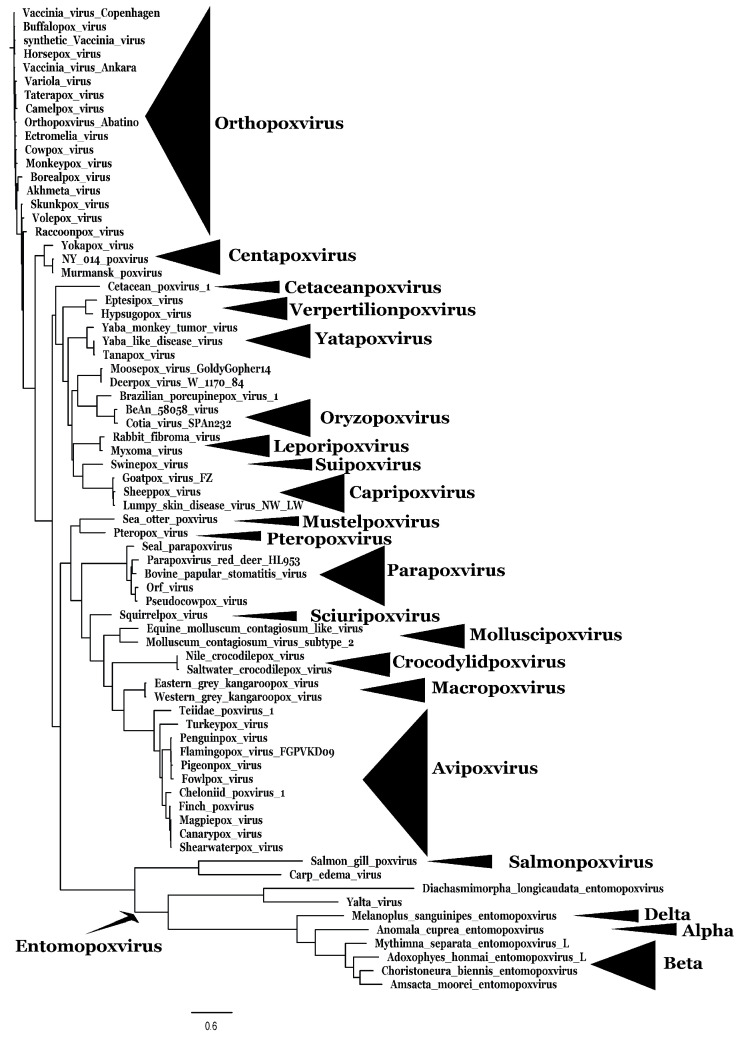
Poxvirus phylogenetic tree. Phylogenetic relationships using 25 genes conserved across the *Poxviridae* family. Sequences were aligned using MAFFT (multiple alignment using fast Fourier transform). Phylogenetic tree was constructed using IQ Tree 2 and visualized using iTOL (interactive tree of life).

Poxviruses are large DNA genome-containing viruses that can infect Metazoans (vertebrates and invertebrates). Many disparate species, including insects, fishes, kangaroos, crocodiles, seals, birds, bats, cows, horses, pigs, rabbits, and humans, are known examples of poxvirus hosts [1,2,3,4,5,6]. The family *Poxviridae* is divided into two subfamilies, the *Chordopoxvirinae* that infects vertebrates and the invertebrate-infecting *Entomopoxvirinae* (Figure 1 and Table 1) [6]. Chordopoxviruses consist of the genera *Avipoxvirus* (poxviruses that infect birds), *Capripoxvirus* (poxviruses that infect cattle, sheep, and goats), *Centapoxvirus* (poxviruses associated with rodents), *Cervidopoxvirus* (poxvirus infecting mule deer), *Crocodylidpoxvirus* (poxviruses infecting crocodiles), *Leporipoxvirus* (poxviruses infecting lagomorphs (rabbits and hares) and squirrels), *Macropoxvirus* (poxviruses infecting kangaroo), *Molluscipoxvirus* (poxviruses infecting humans, chimpanzees, and donkeys), *Mustelpoxvirus* (poxviruses infecting sea otters), *Orthopoxvirus* (poxviruses infecting a wide range of mammals, including primates and rodents), *Oryzopoxvirus* (poxviruses isolated from sentinel mouse), *Parapoxvirus* (poxvirus infecting cows, goats, and gray seals), *Pteropoxvirus* (poxviruses infecting the Australian little red flying fox), *Salmonpoxvirus* (poxvirus infecting Atlantic salmon), *Sciuripoxvirus* (poxviruses infecting red squirrels), *Suipoxvirus* (poxviruses infecting swine), *Vespertilionpoxvirus* (poxvirus infecting the North American brown bat), and *Yatapoxvirus* (poxvirus infecting primates (monkeys and baboons)) [6]. Entomopoxviruses consist of the genera *Alphaentomopoxvirus* (poxviruses infecting insects from the order Coleoptera (beetles)), *Betaentomopoxvirus* (poxviruses infecting insects from the order Lepidoptera (butterflies and moths)), *Deltaentomopoxvirus* (poxviruses infecting insects from the order Orthoptera (North American migratory grasshopper)), and *Gammaentomopoxvirus* (poxviruses infecting insects from the order Diptera) [6].

Poxviruses, particularly members of the *Orthopoxviridae* family, are the causative agents of diseases in humans, primates, rodents, and livestock. Notable human diseases caused by members of poxviruses are smallpox (caused by the variola virus), mpox (caused by the monkeypox virus), tanapox (caused by the Tanapox virus), and molluscum contagiosum (caused by the molluscum contagiosum virus) [3,4,6,7,8]. Among these diseases, smallpox alone has plagued humanity for millennia, killing hundreds of millions of people until it was eradicated in 1980 by a global vaccination campaign. Although it has existed in humans for thousands of years, the exact origin of the variola virus is still unknown. Mpox, on the other hand, can occur in humans and other animals due to the existence of reservoir animals in African countries. The recent 2022-23 global outbreak (122 countries) of mpox caused infection in thousands of individuals, killing more than 300 people [9]. Unlike mpox, tanapox is a rare zoonotic poxvirus disease for which non-human primates are predicted to be the main reservoirs [10]. Similarly to humans, a diverse range of pathogenicity to other animals is observed among different members of poxviruses.

Myxoma virus (MYXV) and Shope fibroma virus (SFV), also known as rabbit fibroma virus, are members of the *Leporipoxvirus* genus that affect lagomorphs from the family Leporidae. The order Lagomorpha comprises the families Ochotonidae and Leporidae, which diverged between 30 and 55 million years ago (Mya) [11]. The family Leporidae includes eleven genera, among which *Oryctolagus*, *Lepus*, and *Sylvilagus* are the best-studied and are estimated to have diverged approximately 12 Mya [12]. MYXV and SFV are naturally found in the American rabbits *Sylvilagus brasiliensis* and *Sylvilagus bachmani*, respectively [13]. In these American leporid species, MYXV causes a benign cutaneous fibroma that rarely progresses to other tissue sites [14]. However, in European rabbits (*Oryctolagus cuniculus*), MYXV causes a systemic lethal disease called myxomatosis, characterized by conjunctival inflammation, elevated rectal temperature, swollen lesions at the site of virus inoculation, wide dissemination via the lymphoreticular system, and secondary lesions on the ears and eyes [15,16,17,18,19]. Due to the lethal phenotype of MYXV in rabbits, it was introduced in Australia and Europe in the early 1950s to control the feral European rabbit population, inadvertently starting a continent-wide experiment of natural history and evolutionary adaptation to re-define a mammalian host–pathogen interaction [20]. However, in both continents, there was subsequent coevolution of both MYXV and rabbits. Over time, viral evolution and host resistance led to a balance. In this case, attenuated mutants of MYXV were naturally selected, because the infected rabbits survived longer, and attenuated viruses could be defeated by the rabbits which evolved immune defenses [13]. Another example of host adaptation shaping poxvirus evolution involves the necroptosis pathway of leporids (and cetaceans). Indeed, a strong correlation has been observed between the disruption of necroptosis in these hosts and the absence of the N-terminal domain of E3-like homologs responsible for necroptosis inhibition in the naturally infecting poxviruses [21]. Necroptosis is an evolutionary form of programmed necrosis that contributes to the innate immune response by killing pathogen-infected cells. Moreover, the analysis of rabbit exomes revealed a clear pattern of parallel evolution, where selection acted on standing genetic variation favoring the same alleles in Australia, France, and the United Kingdom. Many of these changes occurred in immunity-related genes, supporting a polygenic basis of genetic resistance [22].

The European rabbit has gained increasing recognition as an animal model for studying a wide range of human diseases [16]. Notably, based on the genetic distance to human genes, the European rabbit is a more suitable model than the mouse to study genes of the innate immune system [23]. The study of MYXV has been important for the ongoing development of MYXV as a potential oncolytic virotherapeutic for the treatment of a variety of human cancers by exploiting the ability of the virus to productively infect a wide diversity of non-rabbit cancer cells [24].

The virion architecture of poxviruses is large, enveloped, and brick-shaped, ranging from 200 to 400 nm in dimension [1,6,25]. Poxviruses contain a linear double-stranded DNA genome that can vary between 100 and 450 kbp in size [25]. At the viral attaching and binding level, poxviruses do not rely on any particular viral or host surface molecule for virus entry [1,26]. Instead, they utilize highly ubiquitous and conserved cell surface proteins like glycosaminoglycans (GAGs) in vertebrates and invertebrates [1,27,28].

After poxvirus attachment, the virion is then internalized to the cytoplasmic milieu by fusing to the plasma membrane or is endocytosed within vesicles [29,30]. Unlike most other DNA viruses, poxviruses replicate entirely in the cytoplasm of infected cells in inclusions referred to as viral factories or virosomes [31]. The infectious virion packages the polymerases and transcription factors required for viral early gene expression.

The genomes of poxviruses are organized such that the genes at the center of the genome are involved in core functions such as transcription, translation, and virus morphogenesis [32,33]. In contrast, genes located at or near the termini modulate the infected host cell in a myriad of ways to allow permissive virus replication in the face of endogenous innate and intrinsic pathways designed to protect the cell from an infection. Furthermore, poxviruses express the genetic information required to amplify the incoming virus in three distinct phases: early, intermediary, and late gene expression [34,35,36,37,38,39]. Prior to early gene expression, encapsidated factors (mostly found within lateral bodies of the virion) are quickly released into the cytosol to start the cell takeover machinery. Next, in the early phase of virus gene expression RNA polymerases, immune modulators, and viral products involved in intermediary gene expression are the first to be synthesized. The intermediate gene expression follows, where viral genes such as late transcription factors and host immune modulators are expressed. The final cascade of virus late gene expression includes proteins involved in virus morphogenesis, early transcription factors to be encapsidated for the subsequent round of virus infection, and host immune modulators [33,34,37,38,39]. Lastly is infectious virion production; the extracellular enveloped virions (EEV) and the intracellular mature virions (IMV) are two well-characterized infectious virion types produced that can continue the infection and replication cycle [40,41].

This review focuses on studied host immune modulators (viral proteins that regulate host immune system function for infection, replication, and pathogenesis) and host range factors (viral proteins that determine which host species a virus can infect and replicate within) of Leporipoxviruses, particularly MYXV, and how new leporid poxviruses might arise and jump into new species. In the extracellular environment, poxviruses can bind to and enter most cell types. Once within the cells, the intracellular environment determines whether the infection will be abortive or productive. As a result, virus binding and entry should not be the primary determinant of poxviruses that probe to infect new host species. The intracellular environment becomes the site where virus–host interactions restrict poxviruses–MYXV–to certain species. A summary of all studied immune modulators in MYXV known to date and their effects on virus virulence will be addressed in the context of the host cell immune environment. Lastly, we address some poxvirus genome architectural features that provide the basis for the innovations and adaptations that mediate host tropism and species leaping.

## 2. The Poxvirus Extracellular Environment

### 2.1. Virus Attachment

Poxviruses are promiscuous in terms of binding and entering cells from different animal species and tissue lineages. At the cellular surface environment, the artillery of viral and host proteins regulating virus attachment and entry seems to be highly conserved amongst all poxviruses and diverse animal species [42]. Poxviruses bind ubiquitously expressed cell surface proteins such as heparin and chondroitin sulfate on most animal cell surfaces [28,29,30,43]. 

Four viral proteins have been identified in vaccinia virus (VACV), the best-studied poxvirus model, which mediate attachment of the mature virion (MV) to the cells. Vaccinia virus Western Reserve (VACV_WR) *D8L* (VACV-WR_113), *A27L* (VACV-WR_150), *H3L* (VACV-WR_101), and *A26L* (VACV-WR_149) are directly involved in virus attachment [44,45,46,47,48,49]. The viral H3 protein is highly conserved amongst poxviruses and binds to heparan sulfate on cell surface receptors [47]. A mutant vaccinia virus lacking the H3 protein is defective in progeny virion formation and is not lethal to inoculated mice [47,50]. It should also be noted that the H3 protein of VACV is a primary target for neutralizing antibodies in humans [51]. Another viral attachment protein, A26, binds to cell surface laminins [48,52]. In addition, the viral D8 protein binds to chondroitin sulfates in cells to facilitate viral adsorption [45,49]. In viral experimental assays, mutant VACV missing the *D8L* gene poorly bound to cells and soluble purified D8 protein could interfere with wild-type VACV adsorption, suggesting a role of D8 protein in poxvirus binding/entry [45]. Chung et al. demonstrated that VACV protein A27 is bound explicitly to heparan sulfate molecules of glycosaminoglycan (GAG) during virus infection [46]. Using isolated heparin protein, the authors further demonstrated the effect of heparin in blocking poxvirus infections from diverse families—cowpox virus, rabbit poxvirus, myxoma virus, and Shope fibroma virus [46]. The viral attachment proteins are multifunctional, for example, the H3 protein also participates in MV assembly [47].

Myxoma virus encodes orthologues to VACV attachment proteins—M083 (D8), M115 (A27), and M071 (H3). Of these, M083 of MYXV has been studied the most [53]. Myxoma virus recombinant construct lacking the *M083L* gene is not defective in virus replication [53]. Nonetheless, the virus is defective in spreading from epithelial to primary lymphocytes, suggesting that the M083 protein is required for virus dissemination via immune cells [53].

The presence of orthologous attachment proteins in MYXV and VACV suggests a possible conserved mechanism of poxvirus attachment shared by all extant poxviruses. Since the viral and host attachment machinery mediating poxvirus cellular contacts is most likely conserved, it seems reasonable to suggest that most poxviruses that encounter cells from novel host species ($S_{n}$) will not have a problem attaching/binding to them. In addition, if the natural host species cells ($S_{h}$) and $S_{n}$ share conserved evolutionary surface molecules needed for virus attachment, it can be reasoned that $S_{h}$~$S_{n}$. In such a model in which both $S_{h}$ and $S_{n}$ are similar, a poxvirus venturing to a new species would encounter few constraints in viral attachment. 

### 2.2. Virus Fusion and Entry

To address the idea of poxvirus fusion and entry, we pose the following questions: Does poxvirus fusion and entry also obey the same prediction of robustness as binding/attachment? Which system is better suited for endowing cellular promiscuity, a single viral protein performing the task, or multiple viral proteins working together on the same task? Unlike most other enveloped viruses that mediate viral fusion/entry with one to two proteins, poxviruses are particularly remarkable in that, at least for VACV, at least 11 proteins mediate viral entry. The entry-related proteins are required for poxvirus entry/fusion of both the mature and the extracellular virions (EVs). Strikingly, most viral proteins mediating entry are conserved across all poxviruses. The following summary briefly introduces the poxvirus entry proteins and some biology. A more detailed review of poxvirus-related attachment and entry proteins can be found in Moss et al. 2012 and 2016 [29,30].

Vaccinia virus A16 is a transmembrane domain-containing protein present in the mature virion. Efforts to isolate the VACV A16 null virus were unsuccessful, suggesting that *A16L* gene products are required for a successful virus life cycle. Furthermore, *A16L*-deficient virus bound to cells but the viral cores failed to penetrate the cytoplasm [54]. Another entry-related protein, viral *F9L* gene product, is a conserved poxvirus protein that induces neutralizing antibodies to VACV infection [55]. The L1 is also a cellular entry protein similar to the F9 protein [56]. Eight other VACV-related proteins are critical components of virus–cell fusion (A21, A28, G3, G9, H2, J5, L5, and O3) [57]. Mutant viruses lacking any fusion-related proteins fail to successfully complete the virus life cycle and produce progeny virions. Viral fusion proteins are conserved amongst all poxvirus, as with virus attachment-related proteins [29,30].

DELTA BLAST (Domain Enhanced Lookup Time Accelerated Basic Local Alignment Search Tool) (https://blast.ncbi.nlm.nih.gov/Blast.cgi (accessed on 9 August 2025)) using known fusion proteins from vaccinia viruses as queries against myxoma viruses reveals the following orthologues: A16 (M105), F9 (M019), L1 (M055), A21 (M110), A28 (M116), G3 (M046), G9 (M054), H2 (M070), J5 (M067), L5 (M059), and O3 (M037) in myxoma virus [35].

Assuming the minimum requirements for poxvirus entry are conserved across poxviruses and diverse cellular lineages, it is reasonable to hypothesize that any live recombinant poxvirus will also encounter few constraints in binding and entering cells from diverse species. 

## 3. The Poxvirus Intracellular Environment

Poxviruses are unique amongst DNA viruses in that they replicate entirely in the cytoplasm of infected cells. Within the intracellular milieu, poxviruses must overcome a barrage of innate immune-related factors expressed either constitutively or inducibly by the host. For example, host cells can activate necroptosis cell death and induce interferon production to circumvent virus replication and spread [58]. If host defenses were always successful in eliminating invading viruses, one would expect all viral lineages infecting a given species to have gone extinct. This, however, can only be one side of the evolutionary interplay, as viruses also can mount an anti-host response to inhibit or circumvent the cellular innate immune pathways. Poxviruses are master immune modulators that encode many dozens of proteins to thwart the host’s self-protective immune responses. About half of the poxvirus genome encodes genes that modulate the host’s innate immune system for successful virus replication and progeny virion formation [2,59,60,61]. For example, MYXV encodes the M029 protein (orthologue of the orthopoxviral E3 family of proteins) responsible for antagonizing host PKR (protein kinase R) activation for successful virus replication [62,63]. The summated tug of war between the varied host and viral immune-related proteins shapes the molecular immune environment between viruses and their hosts. 

Unlike bacteria that primarily utilize nucleic acid-mediated host cell defenses against invading viruses, i.e., CRISPR (Clustered Regularly Interspaced Short Palindromic Repeats), metazoans and land plants can further exploit a protein-mediated defense to counteract virus infections. The immune composition of metazoans encompasses innate and adaptive immune systems, with the adaptive immune system unique to jawed vertebrates [64,65]. The innate immune system recognizes conserved pathogen-associated molecular patterns (PAMPs) like non-self epitopes and double-stranded RNA (dsRNA). In contrast, the adaptive immune system is tailored to a particular microbial infection and has a memory component associated with it [66,67].

Although present in all metazoans, the innate immune system-related proteins do not always share significant sequence similarity between species and presumably mediate nonidentical functions. For example, PKR, a central host sensor molecule that shuts down protein translation during virus infection, is not entirely conserved amongst jawed vertebrates. Some organisms are missing key functional domains of this protein kinase [68], raising the question of whether these organisms combat viral infections in the same mechanistic fashion. One can hypothesize that they encode a protein that is related to, yet distinct from, the canonical human PKR, potentially having a similar though not identical function. If this model is accurate then the function of PKR as an innate immune modulator in jawed vertebrates is not wholly conserved and thus viral anti-PKR strategies might also be species-specific. The innate immune system, therefore, can be viewed as an environment shaped by species-unique evolutionary processes, likely shaped by ancestral pathogen challenges [68].

Additionally, we can expect that such species-specific immune-related selection pressures have also shaped their respective infecting viruses, creating a virus–host species-specific mutualist environment. In this model, no two species’ immune systems are interchangeable because they are tailored for that species. A similar case can be made that no two poxviruses’ immune modulators are interchangeable, particularly if they normally infect different host species. But is this always true? With current technology, transplanting species’ entire innate immune systems would be difficult, if not impossible. Nonetheless, we can ask more modest questions using viruses. Mainly, can a virus adapted to species $S_{1}$ be modified to infect and cause disease in species $S_{2}$? Empirical data from poxviruses that leap into new host species can serve as a litmus test for such ideas. 

A case can be made by considering a theoretical all-encompassing immune system (AEI) for known viral pathogens. The idea is that if such an all-encompassing immune system ($I_{e}$) existed, other less vulnerable immune systems$-I_{x}$—are contained within $I_{e}$. It should hold that $I_{e}$ can protect organism (X) containing an immune system $I_{x}$ since $I_{x}$ is a subset of $I_{e}$. Assuming two poxviruses $P_{e}$ and $P_{x}$, which evolved to counteract the immune environments of $I_{e}$ and $I_{x}$, respectively, then virus $P_{e}$ should be permissive to $I_{x}$ while $P_{x}$ should be restricted to $I_{e}$, following the AEI line of reasoning. Since anti-immune gene replacement experiments are impossible, can poxviruses be used to test the AEI idea? If so, how do we ask the questions and interpret the results? 

Alternatively, closely related species may share similar basic biological features of their innate immune system $I_{e}$~$I_{x}$. In other words, viruses infecting these species would navigate a similar immune environment. The prediction would be that since $I_{e}$~$I_{x}$, $P_{e}$ and $P_{x}$ should be permissive within the context of the closely related immune environments.

The next section will discuss our knowledge of studied MYXV immune modulators and their impact on virus fitness and pathogenesis. Our aim is to synthesize the molecular mechanisms of the known MXYV immune modulators and their roles in virus pathogenesis (myxomatosis in rabbits and after species leaping into hares). Furthermore, we hypothesize about some uncharacterized molecular mechanisms of action of described MYXV immune modulators to address the gaps in our knowledge.

## 4. Immune Modulators Located Within the MYXV Genome Terminal Inverted Repeats (TIRs) or Duplicated Copies of Immune Modulator Genes

### 4.1. M001R/L (M-T1)

**Figure 2:**
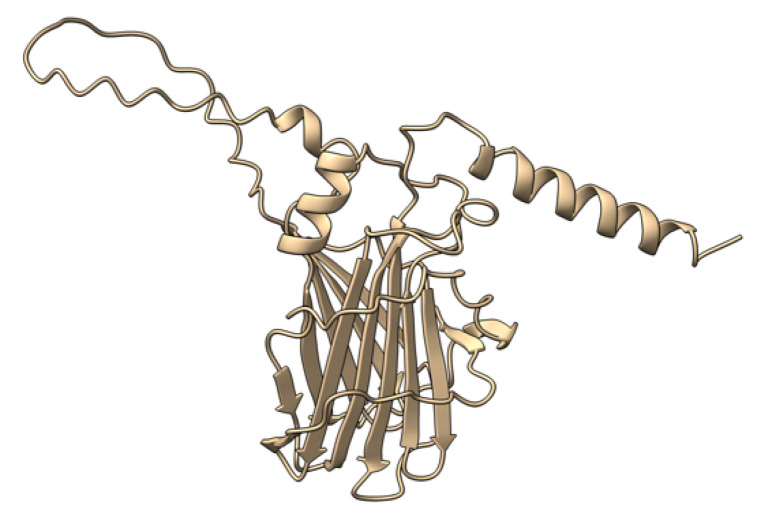
Predicted alpha fold structure of T1.

Myxoma virus *M001R/L* (also called M-T1, or simply T1) encodes a 260 aa protein (Figure 2) that is synthesized and secreted early during virus infection to modulate the host immune response [69]. Extracellular T1 protein binds to rabbit and human CC chemokines, including RANTES (regulated upon activation, normal T-cell expressed and secreted) and IL-8 (interleukin 8), modulating immune cell recruitment to the tissue sites of virus infection [70]. RANTES plays a role in the recruitment of T-cells, macrophages, and myeloid-derived cells to sites of infection and inflammation. IL-8 promotes the migration of neutrophils and angiogenesis at same sites. Although targeted deletion of both copies of the *M-T1* genes did not significantly affect European rabbits’ disease progression and mortality rate, the influx of monocytes/macrophages significantly increased into lesions caused by the *M-T1* deficient virus compared to the wild-type virus [69]. Molecular and cellular data suggest that *M-T1* restricts migration of monocytes and macrophages into virally infected tissues to reduce virus detection and promote infection and dissemination [70,71].

### 4.2. M002R/L (M-T2)

**Figure 3:**
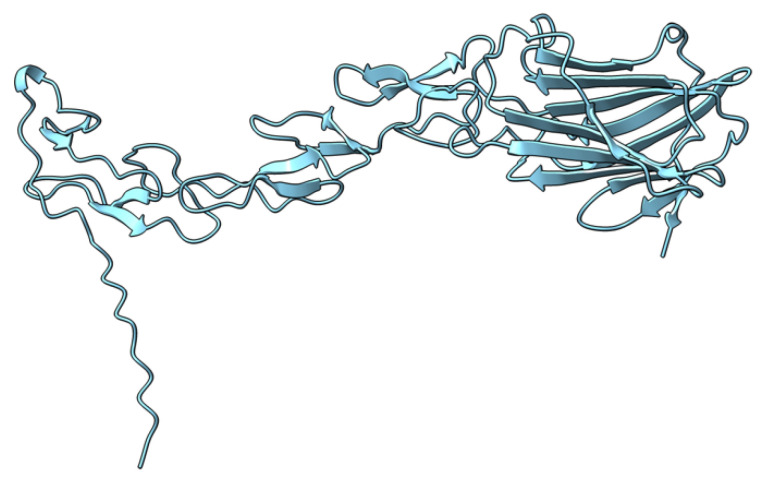
Predicted alpha fold structure of T2.

Myxoma virus *M002R/L* (also called M-T2 or T2) encodes a 326 aa protein (Figure 3) expressed and secreted early during virus infection. T2 shares sequence and structural homology to the cellular tumor necrosis factor (TNF) receptor (TNFR) [72,73,74]. During MYXV infection, T2 is secreted to counteract rabbit TNF, both as a monomer (55–59 kDa) and a disulfide-linked dimer (90 kDa). Both forms have been shown to bind rabbit TNF, although the dimeric form is more potent as an inhibitor [75,76]. However, T2 cannot bind or inhibit human and mouse TNF, suggesting that it is adapted to antagonizing only rabbit-derived TNF. Intracellular T2 is also an immunomodulator with a separate target, the PLAD domain of TNFR, critical for inhibiting apoptosis in rabbit lymphocytes. Interestingly, this intracellular anti-TNFR property of T2 is not species-specific, suggesting that the cellular receptor is structurally more species-conserved than the ligand. European rabbits infected with T2-deficient MYXV successfully mounted an antiviral response against the virus [73]. Rabbits that survived T2 knockout virus infection recovered and later survived re-challenge with wild-type MYXV [73]. The preceding ideas suggest that the myxoma virus *M002R/L* gene product is an immunomodulatory protein critical for successful virus pathogenesis.

### 4.3. M004R/L (M-T4)

**Figure 4:**
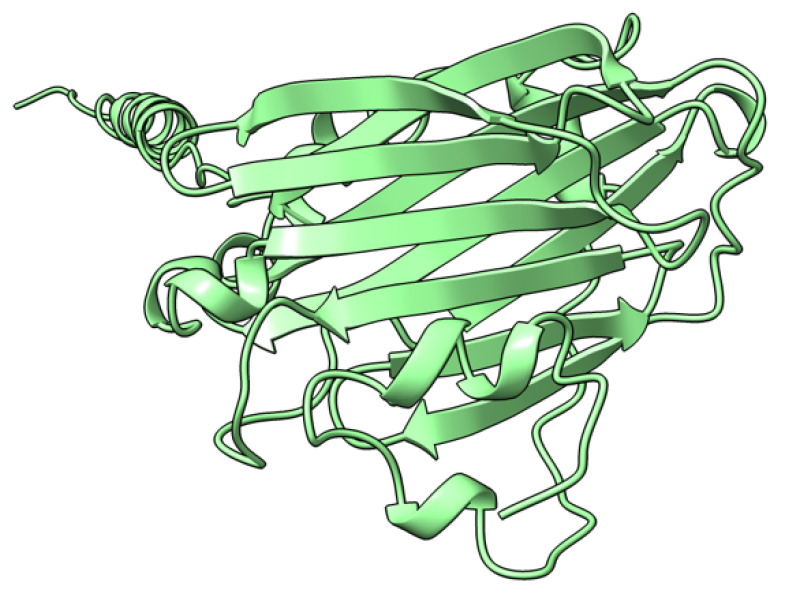
Predicted alpha fold structure of T4.

M-T4 (or T4) is a 237 aa intracellular protein (Figure 4) with a C-terminal ER (endoplasmic reticulum) retention signal (-RDEL) produced early during virus infection. M-T4 predominantly co-localizes with calreticulin, suggesting T4 protein is primarily retained in the ER [77]. Further work demonstrated that the -RDEL motif did not affect T4 localization to the ER, although the protein stability was affected [77]. T4-deficient MYXV induced rapid apoptosis in rabbit peripheral blood mononuclear cells (rPBMCs) and RL5 lymphocytic cells, suggesting a role of T4 in counteracting virus-triggered cell death [78]. The RDEL-minus-T4 mutant MYXV activated apoptosis in RL5 cells, although to a lesser extent than the complete T4-knock-out (KO) virus [78]. In European rabbit pathogenesis studies, T4-KO-infected rabbits had a lower virulence than rabbits infected with the wild-type MYXV strain. However, the RDEL mutant T4 virus exhibited a unique myxomatosis disease phenotype characterized by excessive edematous and inflammatory responses at secondary sites of infection [78].

### 4.4. M005R/L (M-T5)

**Figure 5:**
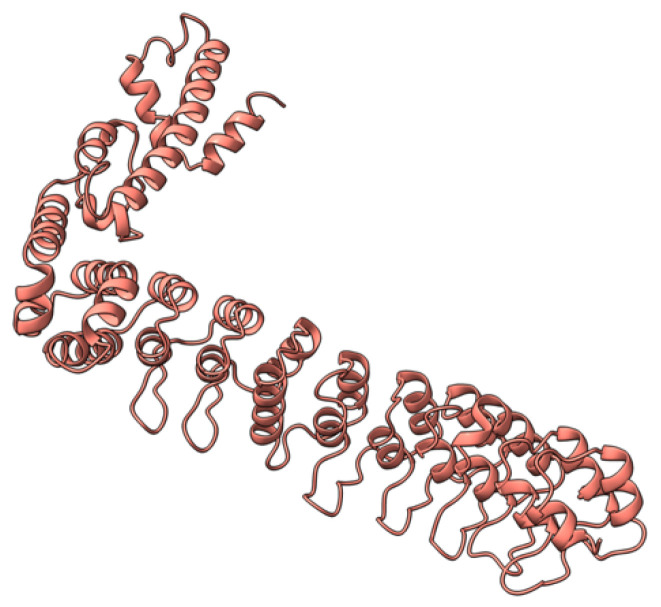
Predicted alpha fold structure of T5.

M-T5 (T5) protein is an intracellular 483 aa ankyrin repeat-containing (ANK-R) protein (Figure 5) synthesized early during virus infection. T5 is one of the four ANK-R containing proteins encoded by MYXV. T5 molds the intracellular milieu to promote virus infection [79], and binds host protein kinase B (Akt) and Skp1, reprogramming their activity during successful virus replication [80,81,82]. MYXV infection lacking both copies of the T5 gene resulted in cells being arrested at the G_0_/G_1_ phase and increased levels of p27/Kip-1 proteins [83]. Infection of rabbit RL5 cells and rabbit PBMCs (rPBMCs) with M-T5-KO MYXV resulted in rapid cell death and a significant drop in the viral titers. European rabbits infected with T5-minus MYXV did not show signs of classical myxomatosis [84]. The virus could not spread beyond the primary site of infection, with no signs of systemic virus dissemination [84,85]. The ability of T5 to promote successful disease progression suggests its role as a key innate immune modulator.

### 4.5. M007R/L (M-T7)

**Figure 6:**
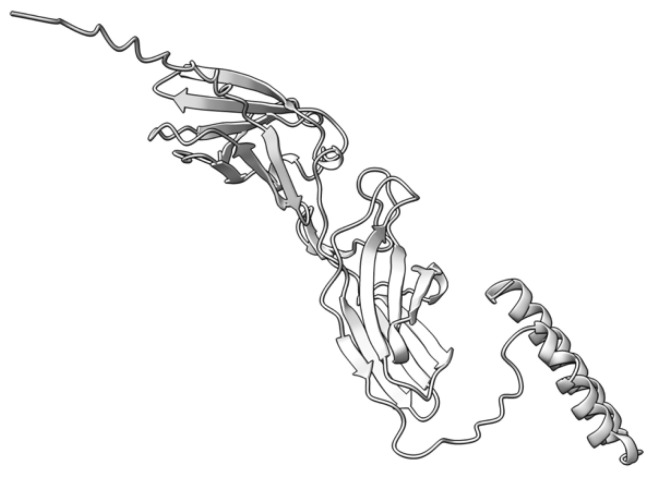
Predicted alpha fold structure of T7.

M-T7 (T7) is a 263 aa secreted protein that acts extracellularly and shares sequence similarity with the interferon-gamma receptor (Figure 6). T7 is secreted at early time points during virus infection and binds/inhibits rabbit IFNγ, plus a wide variety of C-, CC-, and CXC-chemokines to modulate multiple pathways of the immune system [86,87]. Studies with MYXV lacking both copies of the *M-T7* gene demonstrate that T7 is dispensable for MYXV replication in cultured RK13 cells. However, European rabbits infected with MYXV deficient in T7 showed decreased virulence and pathogenesis [88]. The loss of T7 also led to a significant decrease in MYXV spread to secondary sites. Compared to the wild-type virus, rabbits infected with T7-KO MYXV exhibited increased numbers of infiltrating leukocytes at the primary site of infection and increased lymphocyte activation in the spleen and lymph nodes [88]. This suggests that T7 is a potent immune modulatory protein that regulates the localization of leukocytes in virally infected tissues.

### 4.6. M008.1 (Serp-1)

**Figure 7:**
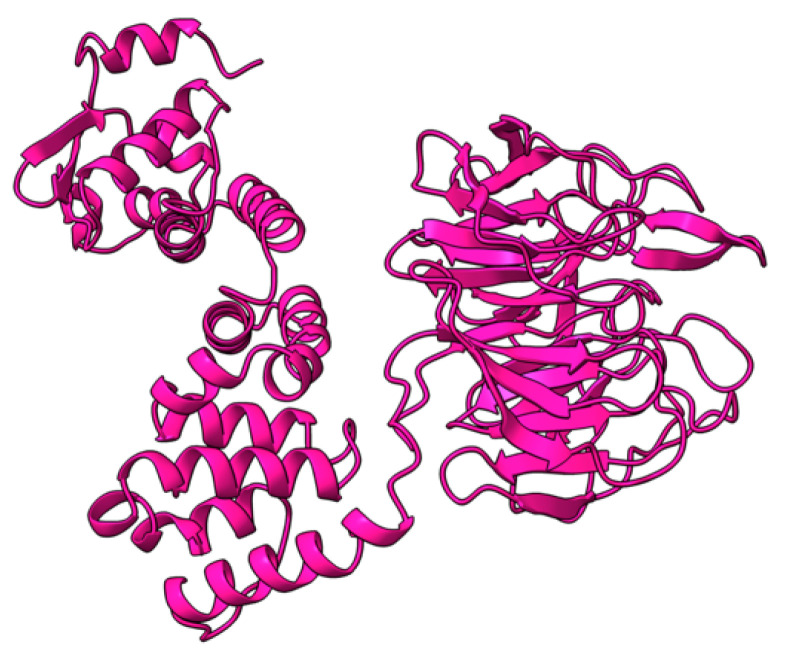
Predicted alpha fold structure of Serp-1.

Serp-1 is a 369 aa secreted viral protein (Figure 7) that is unusual in that it is expressed at late times of viral infection and modulates inflammatory myeloid cells around the virally infected tissues. Like other secreted MYXV proteins, Serp1 is glycosylated by cellular enzymes during infection [89,90]. In addition, Serp-1 deletion does not affect myxoma virus replication in cultured RK13 cells, suggesting that it is not required for successful virus replication and virion assembly. In European rabbits, infection with MYXV missing both copies of the Serp-1 gene is attenuated. Serp-1 deficient MYXV produced >50% recovery rate in infected European rabbits, suggesting that Serp-1 promotes successful virus immune evasion [91]. Tissue analysis from rabbits infected with MYXV missing Serp-1 showed a more significant inflammatory cell response than the wild-type virus [91]. This indicates that Serp-1 interacts with cellular immune pathway components to modulate cellular inflammation and promote virus dissemination in vivo. Interestingly, Serp-1 protein has been purified and shown to act as a potent anti-inflammatory drug in a wide variety of viral and non-viral inflammatory diseases [92].

## 5. Single Gene Copy MYXV Immune Modulators

### 5.1. M010L (M10)

**Figure 8:**
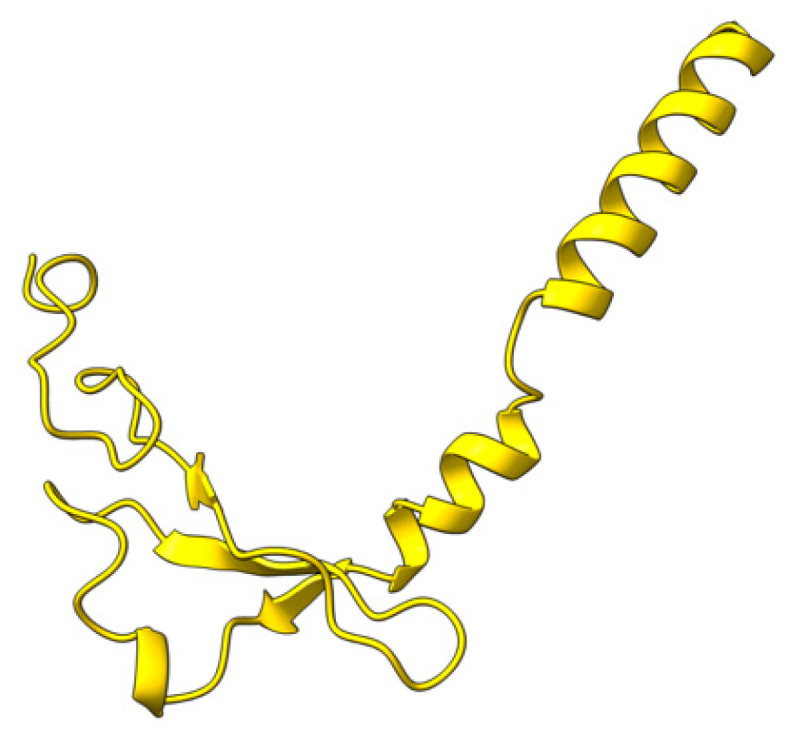
Predicted alpha fold structure of M10.

M10, also known as myxoma growth factor (MGF), is a 65 aa secreted protein synthesized early during viral infection (Figure 8). M10 shares homologous domains with cellular TGFα (transforming growth factor alpha) and acts by binding as a ligand to the epidermal growth factor receptor (EGFR) [93]. There is little information about the intracellular functions of M10. However, MYXV lacking the single copy *M010L* gene resulted in moderated symptoms of myxomatosis in infected European rabbits [94,95]. Furthermore, virus growth assays in cultured rabbit-derived spleen cells showed an average 0.5 log decrease in viral titers for MYXV lacking the M10 gene product compared to the wild-type virus. This indicates that M10 is required for optimal MYXV replication and pathogenesis in rabbits.

### 5.2. M011L (M11)

**Figure 9:**
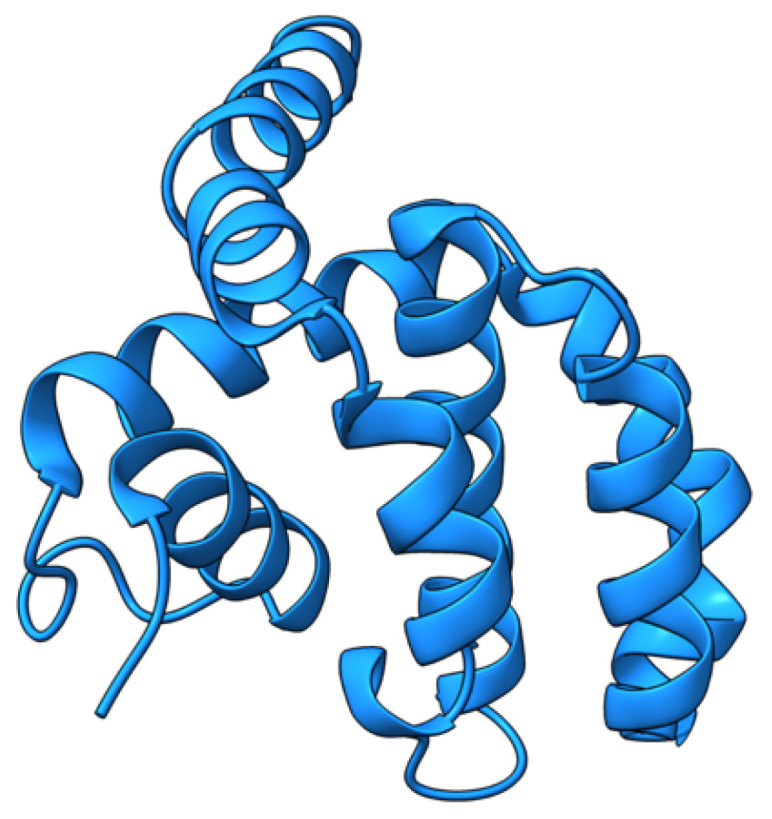
Predicted alpha fold structure of M11.

Myxoma virus M11 is a 115 aa early synthesized intracellular viral protein used to thwart apoptosis induction during virus infection. M11 contains a Bcl-2 domain (Figure 9) and co-localizes with the mitochondria and also other intracellular cell membranes of infected cells [96,97]. The M11-deficient virus led to the activation of apoptosis in different mammalian cells infected with MYXV and a decrease in virus titers compared to the wild-type virus, suggesting a conserved apoptosis pathway for various species. Lack of M11 led to the activation of cellular Bax and Bak, as well as the subsequent cytochrome C release from mitochondria within the cytosol of infected cells [98,99]. European rabbits infected with M11-deficient virus showed reduced virulence and increased survival rates [94,100,101]. The data suggest that M11 is an apoptosis regulator required for optimal virus fitness.

### 5.3. M013L (M13)

**Figure 10:**
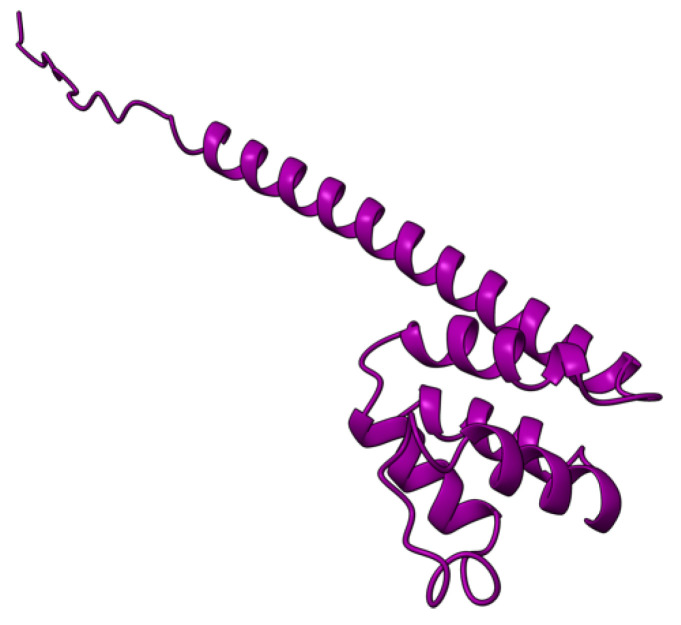
Predicted alpha fold structure of M13.

Myxoma M13 is a 126 aa protein expressed early in virus infection and contains a pyrin domain (Figure 10) that binds to the cellular factor ASC-1 and thus regulates inflammasome activation during the virus infection [102,103]. M13 protein also interacts with NFκB to regulate diverse intracellular pathways, for example, those leading to TNF secretion [104,105]. M13-KO MYXV replication is impaired in RL5 cells and rabbit peripheral blood lymphocytes and monocytes [102]. The absence of M13 mildly affects MYXV progeny virion formation in RK13 cells. This suggests that M13 is a critical immune modulator vital for optimal virus replication in immune cells [102]. Rabbits infected with M13-deficient MYXV exhibited a milder disease compared to the wild-type infected European rabbits [102]. However, the primary site of infection for the M13-knock-out virus became more highly inflamed by day 5 post-infection and recovered, compared to the wild-type virus that exhibited less inflammation followed by increased tissue necrosis [102]. This suggests that M13 is an immune modulator that controls MYXV replication by both regulating the intracellular inflammasomes and extracellular inflammatory networks.

### 5.4. M029L (M029)

**Figure 11:**
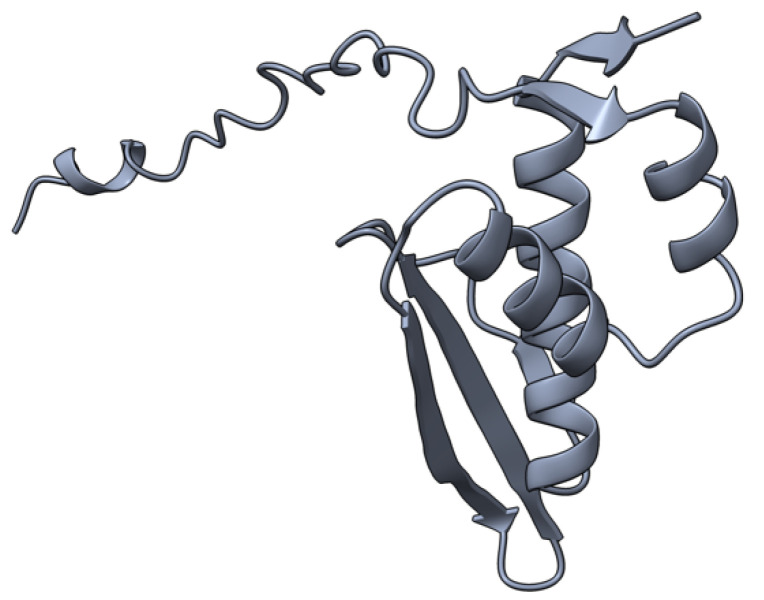
Predicted alpha fold structure of M029.

MYXV M029 is a 115 aa intracellular protein related to the vaccinia E3 regulator. M029 has a canonical dsRNA-binding domain (Figure 11), being synthesized at early time points of viral infection and critical for preventing host translational shutdown during virus replication [62,63,106]. M029 localizes with dsRNA during MYXV infection to prevent the activation of host PKR and translational shutoff. MYXV lacking the M029 gene is severely attenuated in cell lines from diverse species and human cancer cells, suggesting that the M029 immunomodulatory function is universally required for virus survival in cells from multiple species [62]. European rabbits infected with M029-deficient MYXV exhibit essentially no disease compared to the wild-type virus [62]. Furthermore, M029 KO MYXV infection did not protect rabbits after re-challenge with the wild-type MYXV, suggesting that virus infection was aborted quickly before the adaptive branch of the immune system could be engaged.

### 5.5. M062R (M062)

**Figure 12:**
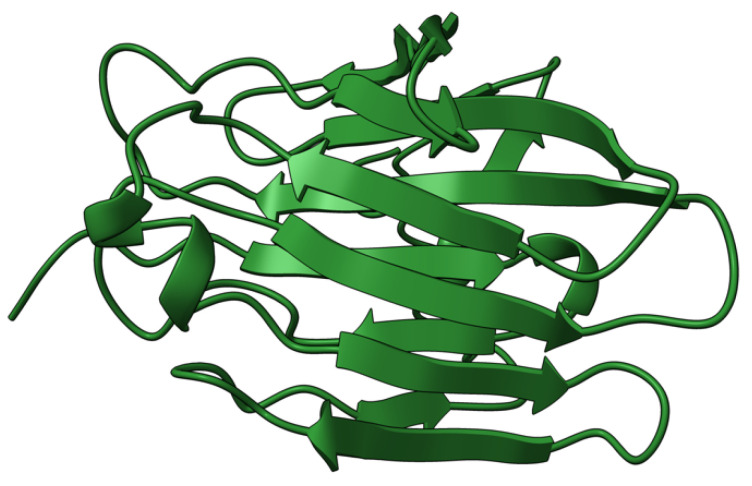
Predicted alpha fold structure of M062.

M062 is a 158 aa intracellular protein produced early and late during MYXV infection (Figure 12). Together with M063 and M064, M062 is a poxvirus C7 host range family member (as defined by the vaccinia C7 gene) critical for successful virus replication [107,108]. These three genes are in tandem in the MYXV genome and likely originated from two distinct duplication events [109]. In rabbit- and human-derived cancer cells, where MYXV can permissively replicate, the lack of M062 leads to a severe attenuation in MYXV growth [107]. Further work demonstrated that M062 binds and antagonizes the cellular interferon-inducible immune regulator SAMD9 to promote virus replication [107,110]. European rabbits infected with the M062-null virus are asymptomatic in terms of disease and are fully protected from re-challenge with a lethal dose of wild-type MYXV [107]. This suggests that M062 is a virus host-range protein critical for antagonizing the host SAMD9 pathway to promote optimal virus fitness.

### 5.6. M063R (M063)

**Figure 13:**
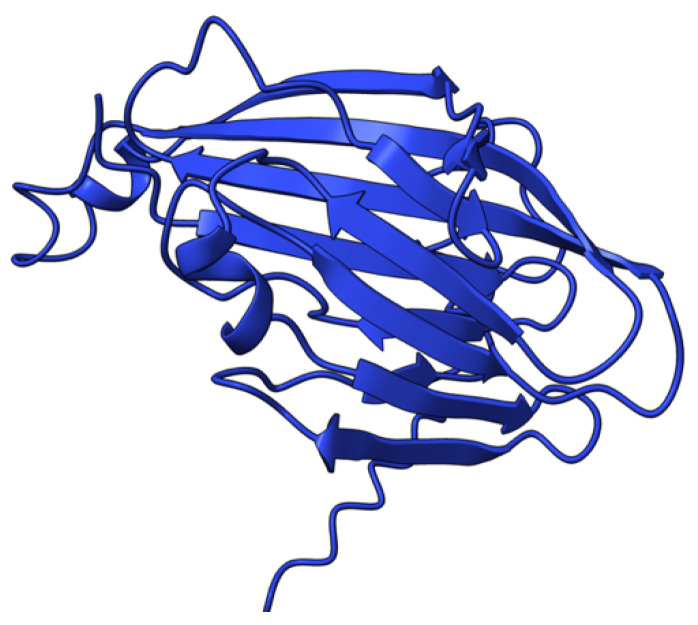
Predicted alpha fold structure of M063.

Like M062, M063 is a 215 aa C7-like family member protein (Figure 13) expressed early during virus replication. In non-rabbit-derived cells permissive to MYXV (such as human or murine cancer cells), absence of M063 did not affect MYXV replication, whereas, in rabbit cells, M063-KO MYXV was utterly defective [111]. European rabbits infected with M063-KO MYXV showed no signs of classical myxomatosis [111,112]. However, re-challenge of recovered rabbits with wild-type MYXV did not progress to myxomatosis [111], suggesting that M063 provided a “vaccine-like” protection effect, even when the virus was not able to replicate. M063 appears to regulate unknown intracellular programs required for optimal virus success in rabbits.

### 5.7. M064R (M064)

**Figure 14:**
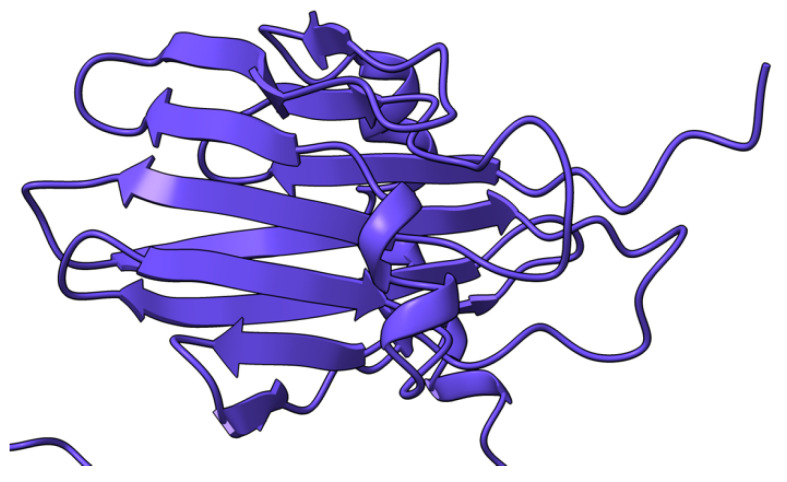
Predicted alpha fold structure of M064.

Myxoma M064 is a 203 aa, C7-like host-range family member protein (Figure 14) that interacts with M063R [113]. Both M063 and M064 are produced at early and late time points of virus infection. In rabbits and non-rabbit-derived permissive cells, the lack of M064 does not affect MYXV replication [113]. In European rabbits, M064-deficient MYXV led to a delay in the progression of classical myxomatosis disease [113]. Although the cellular target of M064 needs to be elucidated, the data suggests that M064 is required for optimal virus pathogenesis.

### 5.8. M128L (M128)

**Figure 15:**
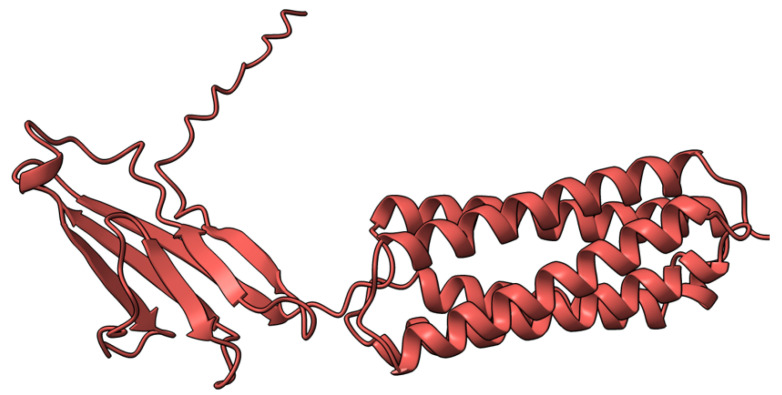
Predicted alpha fold structure of M128.

M128 is a 281 aa membrane protein produced at the late stages of virus infection. M128 is a member of the host CD47 protein family that contains a five-pass membrane-spanning domain (Figure 15) [114]. M128-deficient virus had no defect in growing in tissue culture rabbit-derived cells and had similar titers to the wild-type virus [114]. Rabbits infected with M128-null MYXV did not produce the lethal myxomatosis compared to wild-type viruses [114]. Histological analysis of infected rabbits demonstrated that M128-deficient MYXV recruited a more significant number of activated monocytes and macrophages into lesions than the wild-type virus [114]. This suggests that M128 modulates monocyte/macrophage activation and/or recruitment to promote MYXV pathogenesis.

### 5.9. M130R (M130)

**Figure 16:**
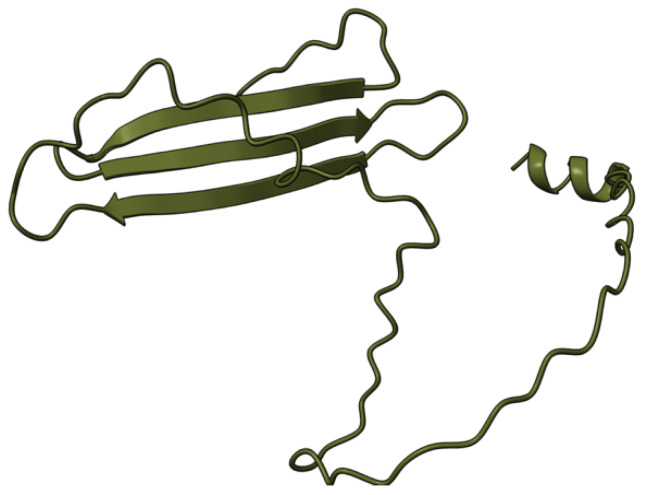
Predicted alpha fold structure of M130.

M130 is a 122 aa presumably intracellular protein (Figure 16) produced late at MYXV infection time points. The *M130R* gene product is dispensable for MYXV replication in rabbits and non-rabbit-derived cells [115]. European rabbits infected with MYXV lacking the M130 can effectively mount an immune response to clear the virus [115]. This suggests that M130 is a viral factor that modulates unknown immune-related pathways to promote virus pathogenesis.

### 5.10. M131R (M131)

**Figure 17:**
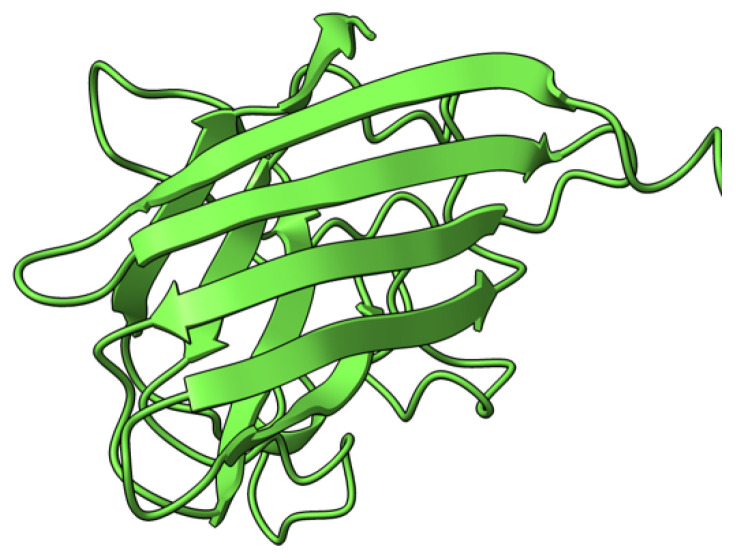
Predicted alpha fold structure of M131.

M131 is a 163 aa intracellular protein synthesized late during virus infection. M131 is a catalytically inactive SOD (superoxide dismutase) family-related protein that does not possess any known dismutase activity of its own (Figure 17) [116]. Unlike most MYXV intracellular immune modulators that are required for optimal virus growth in tissue culture, M131 deletion led to a 10-fold increase in MYXV growth in cultured permissive cells. However, this increase in the M131-deficient virus titers in tissue culture in vitro did not translate to animal experiments in vivo. Although M131-minus MYXV was not used in the animal experiment, SFV lacking the *M131R*-related gene product (called *S131R*) produced smaller fibromas at the site of virus inoculation in European rabbits compared to the wild-type SFV [116,117,118]. Further, molecular studies demonstrated that viral SOD protein binds host copper chaperones, thereby interfering with the proper folding and function of host SODs [116,117]. The data suggest that viral SOD is required for successful virus pathogenesis and may regulate an unknown cellular pathway that limits virus replication.

### 5.11. M135R (M135)

**Figure 18:**
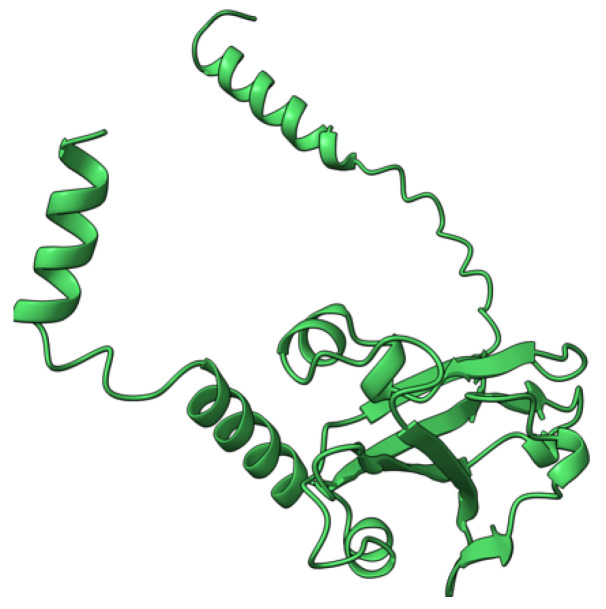
Predicted alpha fold structure of M135.

M135 is a 178 aa early transcribed and expressed viral protein of MYXV that shares sequence similarity with the host interferon-α/β receptor and is thus predicted to bind host IFN α/β to prevent IFN-mediated antiviral pathways (Figure 18) [119]. MYXV lacking the *M135R* gene shows no defect in replication in rabbits or non-rabbit-derived cells whereas deletion of M135 severely attenuates MYXV disease in European rabbits [119]. The data suggest that M135 is an immune modulator targeting an unknown host ligand or pathway critical for MYXV virulence.

### 5.12. M138L (M138)

**Figure 19:**
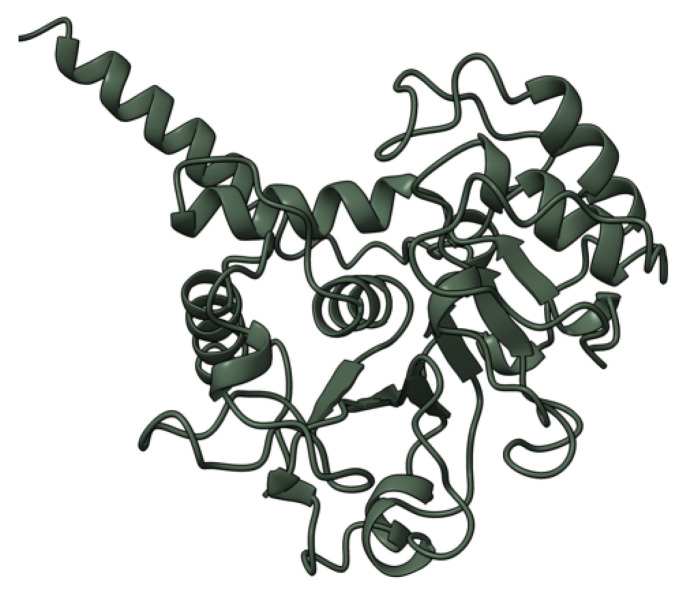
Predicted alpha fold structure of M138.

M138 is a 290 aa protein produced early during virus infection. M138 encodes a sialyltransferase protein (Figure 19) that catalyzes the transfer of sialic acid from CMP–sialic acid to an asialofetuin glycoprotein acceptor, thus affecting the glycosylation of secreted host and viral proteins [120,121]. M138 is dispensable for virus replication in tissue culture. However, unlike the wild-type virus, MYXV lacking the expression of M138 was attenuated in rabbits [121], suggesting that M138 activity is central to successful MYXV pathogenesis.

### 5.13. M141R (M141)

**Figure 20:**
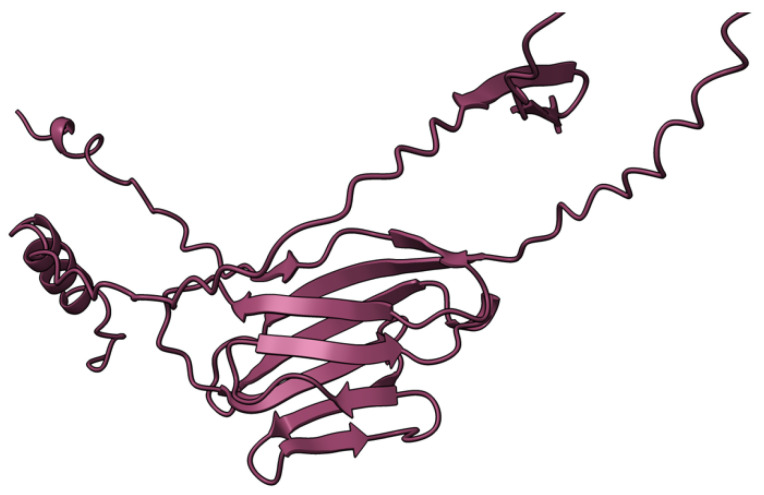
Predicted alpha fold structure of M141.

M141 is a 218 aa early gene product of MYXV expressed on the surface of virus-infected cells. M141 is related to host CD200 (cluster of differentiation) involved in macrophage and monocyte activation (Figure 20) [122,123]. In rabbit-derived cell lines, M141 is dispensable for growth. In rabbits, however, M141 is required for the development of lethal myxomatosis [122]. Tissue analysis of infected rabbits demonstrated greater activation of monocytes and macrophages in rabbits infected with the M141-null virus compared to the wild-type virus [122]. The former idea suggests that M141 is an immunomodulatory protein that dampens monocyte and macrophage activation and/or migration to promote virus pathogenesis.

### 5.14. M148R (M148)

**Figure 21:**
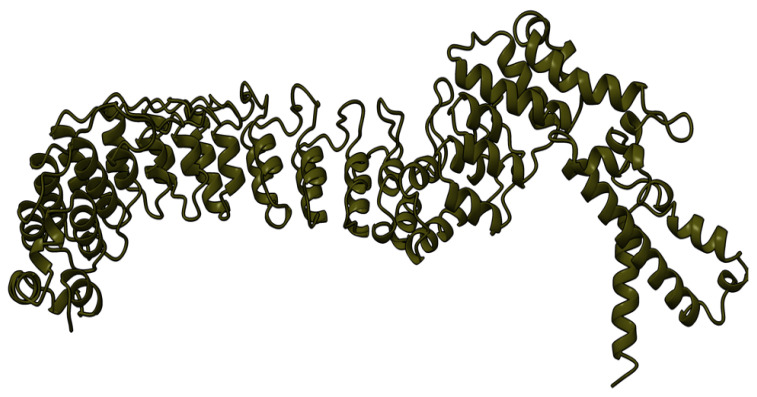
Predicted alpha fold structure of M148.

M148 is a 675 aa late MYXV gene product. Together with M-T5, M149, and M150, M148 forms the poxviral ankyrin-repeat (ANK-R) superfamily (Figure 21). M148 localizes to the nucleolus during virus infection [124]. In rabbits and non-rabbit-derived cells, M148 is dispensable for virus replication. However, in rabbits M148 is required for myxomatosis disease progression, as rabbits infected with M148-null MYXV had higher survival rates than wild-type MYXV [124]. Moreover, rabbits infected with all the ANK-R knockouts (M-T5, M148, M149, and M150) had smaller primary lesions comparing to single knockouts and had an increased activation of the NFκB pathway and release of IL-6 [85]. This shows that the combined functions of these gene products are complex and should be taken into account to fully understand the functional redundancy among these genes.

### 5.15. M149R (M149)

**Figure 22:**
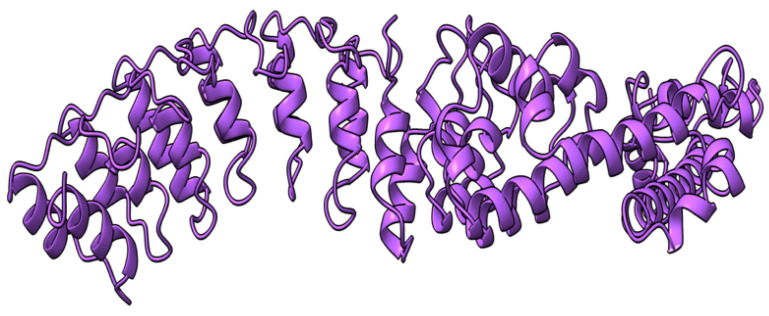
Predicted alpha fold structure of M149.

M149 is a 490 aa early MYXV ankyrin repeat-containing protein (Figure 22) that localizes to the cytoplasm of virus-infected cells [124]. Like M148, M149 is non-essential for MYXV growth in tissue culture, but required for myxomatosis progression in European rabbits [124].

### 5.16. M150R (M150)

**Figure 23:**
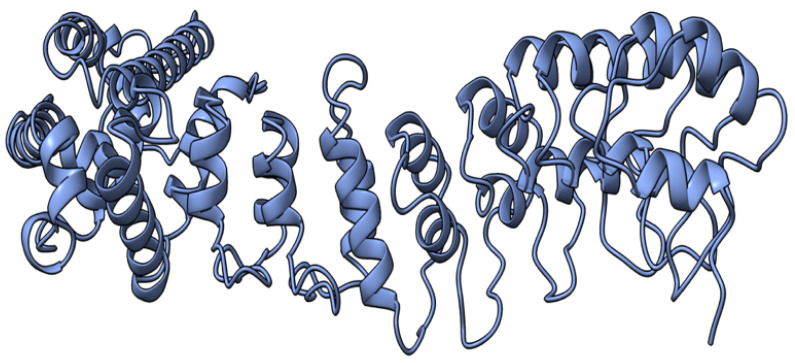
Predicted alpha fold structure of M150.

M150 is a 494 aa early viral ankyrin repeat-containing protein (Figure 23). M150 localizes to the nucleus during virus infection [125]. In cells treated with TNF, M150 colocalizes with host NFκB, suggesting a role in modulating virus-induced signaling linked to inflammation [125]. In rabbit experiments, M150 is a critical virulence factor as the lack of M150 produced a non-pathogenic phenotype compared to the wild-type virus [125].

### 5.17. M151R (Serp2)

**Figure 24:**
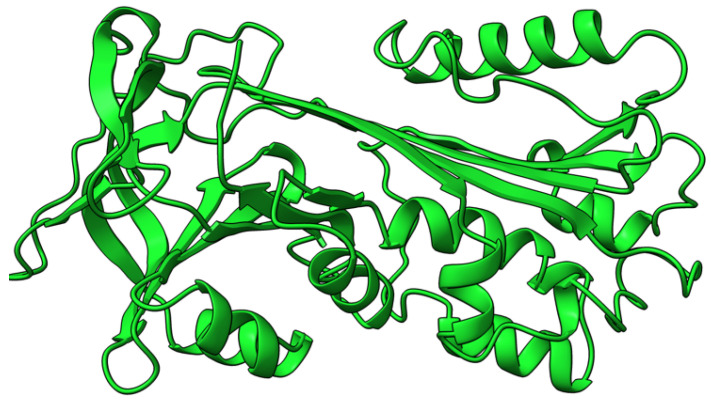
Predicted alpha fold structure of Serp2.

Serp2 is a 333 aa early intracellular viral gene product that binds and inhibits the activity of interleukin-1β converting enzyme (ICE) (Figure 24), thereby preventing the proteolytic processing of pro-IL-1β, an inflammatory cytokine [126,127]. Serp2-null mutant virus replicated to wild-type titers in rabbit-derived cells, suggesting no fitness cost associated with virus replication. In European rabbits, Serp2 is required for the successful progression of myxomatosis [128].

### 5.18. M152R (Serp3)

**Figure 25:**
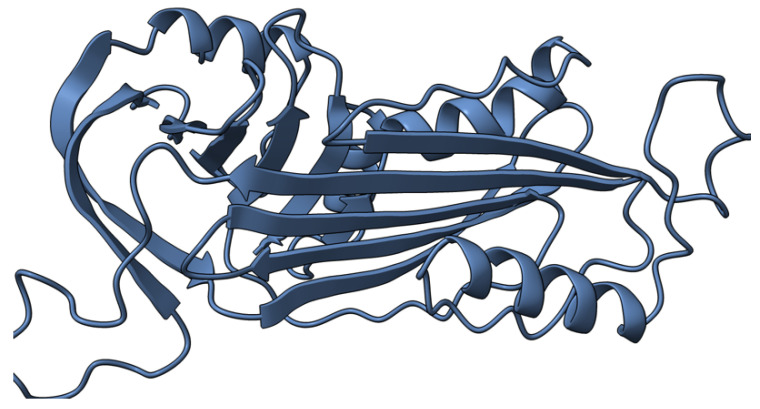
Predicted alpha fold structure of Serp3.

Serp3 is a 266 aa intracellular protein produced at late time points of virus infection (Figure 25). Although Serp3 is dispensable for virus replication in vivo, its deletion hampered viral virulence and dissemination [129]. The data suggest that Serp3 is an immunomodulatory protein required for myxomatosis in rabbits.

### 5.19. M153R (M153)

**Figure 26:**
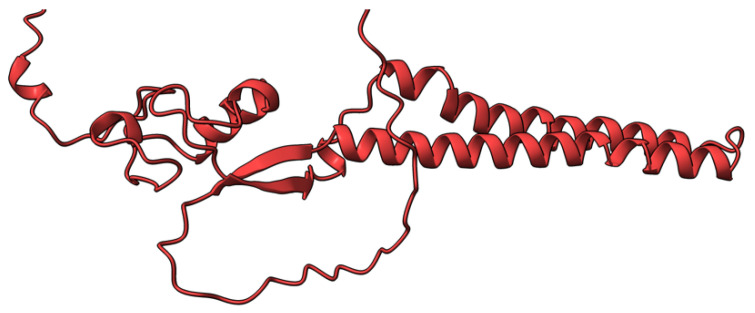
Predicted alpha fold structure of M153.

M153 is a 206 aa E3 ubiquitin ligase-like intracellular protein (Figure 26), produced at early time points of virus infection, that regulates the expression of host cell immune markers such as CD4 and MHC-I (major histocompatibility complex) [130]. M153 downregulates the expression of CD4 and MHC-I by targeting them to the lysosome for degradation, reducing protein availability on the cell surface and viral antigen presentation, thereby dampening the acquired immune response [130,131]. Rabbit experiments demonstrated that M153 is required for the successful progression of acute myxomatosis in European rabbits, suggesting that host targets of M153 may extend beyond those needed for later-stage acquired immunity [131].

### 5.20. M156R (M156)

**Figure 27:**
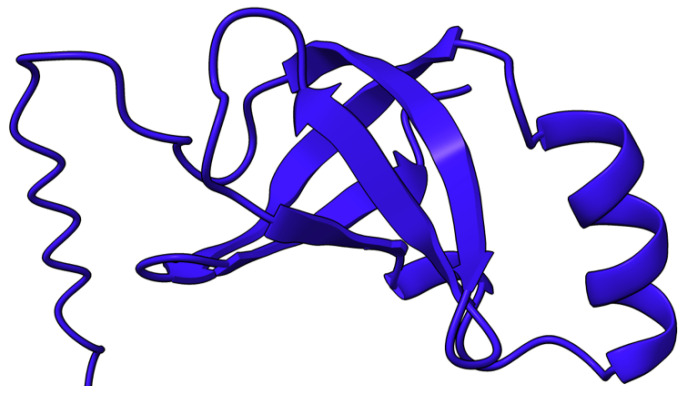
Predicted alpha fold structure of M156.

M156 is a 102 aa early intracellular viral gene product related to the vaccinia K3 regulator (Figure 27). M156 mimics the eukaryotic initiation factor 2 alpha (EIF2α) involved in protein translational regulation. During virus infection, M156 competes for rabbit PKR phosphorylation of EIF2α to prevent protein translation shutdown [132]. M156 is required for virus replication in all rabbit-derived cells tested [132]. Although pathogenesis studies have not been conducted tackling the function of M156 in rabbits, attenuated MYXV field isolates from Australia correlate with mutational loss of M156 [132].

## 6. The Poxvirus Genetic Environment

The mutation rate amongst viruses varies by many orders of magnitude, from $1.5\times 10^{-3}$ mutations per nucleotide per genomic replication cycle (mut/nt/rep) in ssRNA phage Qβ to $1.8\times 10^{-8}$ mut/nt/rep in dsDNA virus herpes simplex virus type 1 (HSV-1) [133]. Poxviruses are thought to have lower mutation rates compared to RNA viruses and adapt to selective forces through a combination of genomic architectural mechanisms. Some genomic mechanisms of poxvirus adaptation include homologous recombination, viral gene duplication, and horizontal gene transfer [32]. Here, we focus on the known genetic mechanisms of adaptation of leporipoxviruses. For a detailed summary of the molecular mechanisms of poxvirus evolution in general, see Brennan et al., 2022 [32].

A seminal paper by Elde et al., 2012, demonstrated that poxviruses can deploy a “genomic accordion” mechanism to adapt to host immune pressures [134]. The idea is that during selection or an attempted host species leap, poxviruses can amplify a maladaptive (or suboptimal) immune inhibitor to counteract the effect of host immune modulators while “sampling” the mutation space for a more adaptive immune inhibitor. The virus’ subsequent gain of an adaptive immune inhibitor is followed by a contraction in the virus genome to offset the cost of a larger genome while maintaining the adaptive variant. The genomic accordion phenomenon suggests a possible mechanistic explanation for poxvirus adaptation while migrating from one host species to another [32,134]. However, the actual intermediate genomic changes are transient and sampling poxviruses in nature in the process of genomic accordion may be difficult and would require regular virus surveillance programs. Yet, it may be relevant that some poxvirus genomes like MYXV and SFV each encode three copies of the poxvirus C7L-like family genes arranged in tandem—MYXV M062, M063, and M064—which possibly arose from two gene duplication events. It is worth noting that all the C7L-like poxviral proteins perform diverse functions, suggesting different operational specialization after duplication and hence their maintenance in the genome [109,135]. Indeed, in myxoma virus, M062 helps to overcome host range restriction by antagonizing SAMD9 activity, with deletion from the MYXV genome resulting in a virus unable to replicate in most mammalian cells [107]. M063, a classic host-range factor, further interacts with M062 and facilitates its binding to SAMD9; its emergence has been suggested to be associated with the unique adaptation of myxoma virus to the rabbit host [109,111]. In contrast, M064 is a virulence factor controlling MYXV infection kinetics and does not present host-range properties as knock-out viruses have no defective host range [113,135]. However, several important questions remain open. Was the origin of the M062, M063, and M064 genes subject to a genomic accordion event(s)? Are poxviruses competitive during the inflationary or contractionary phase of the accordion? Did the accordion event “A” occur during a host species jump by a progenitor virus? What is the poxvirus timescale of gene duplication and specialization events in nature?

Poxviruses can evolutionarily innovate their genome and host-range function by acquiring genes via homologous recombination and LINE (long interspersed nuclear element) retrotransposons [32]. Moreover, poxviruses can acquire new genes from accompanying infecting poxviruses via homologous recombination, thereby innovating their genomes. A notable example includes the species jump of myxoma virus from rabbits to the Iberian hare (*Lepus granatensis*). European rabbits and Iberian hares co-inhabit the Iberian Peninsula in close contact. Since its emergence in the late 1950s, MYXV affected rabbits without causing overt outbreaks of myxomatosis in Iberian hares. In 2018, reports of wild Iberian hares in the Spanish province of Toledo dying of a disease resembling rabbit myxomatosis raised the alarm. These mortalities were linked to a newly isolated strain called MYXV Toledo or hare MYXV [136,137,138,139]. This species jump of MYXV and subsequent disease outbreak across the Iberian Peninsula resulted in an estimated mean mortality rate of 55.4% in hares [137]. Subsequently the hare MYXV was diagnosed in European brown hares (*Lepus europaeus*) and recent reports confirm that the disease is spreading in other European countries like Germany and Netherlands [140]. It is evident from the recent outbreak that the hare MYXV has further evolved and is probably undergoing adaptation in hares. Genomic studies revealed that a MYXV strain extant in southern Europe had acquired a novel gene cassette, possibly derived by recombination from an unsampled poxvirus, allowing it to cross the species barrier from rabbits into hares. The new recombinant MYXV Toledo contained a novel ~2.8 kb region within the *M009L* gene of the MYXV Lausanne strain, encoding four new viral gene products (*M157L*, *M158L*, *M159L*, and *M160L*) [136,138]. Further work demonstrated that M159 is a new member of the poxvirus C7 family of proteins and acts as a host-range protein, being responsible for permitting virus replication in hare-derived cells but not in rabbit cells [141]. The preceding ideas suggest that poxviruses possess diverse genome architectural features that facilitate the biological adaptation needed for successful host species leaping. In leporipoxviruses, recombination and viral gene duplication seem to be responsible for the major evolutionary events leading to host tropism and expansion.

## 7. Conclusions

Poxviruses are diverse and intricate biological machines that encode an impressive repository of immune-related proteins and genomic architectural features. However, given poxviruses can encode more than a hundred different host-interactive factors, most of their functions remain a mystery. The few MYXV immune modulators that have been studied to date educate our fundamental understanding of cellular processes and provide possibilities for novel therapeutic avenues. More work is needed to understand the basic biophysical processes driving poxvirus biology and evolution.

## Figures and Tables

**Table 1:** Taxonomy of the family *Poxviridae*.

Subfamily	Genus	Host(s)
*Chordopoxvirinae*	*Avipoxvirus*	birds
	*Capripoxvirus*	cattle, sheep, and goats
	*Centapoxvirus*	rodents
	*Cervidopoxvirus*	mule deer
	*Crocodylidpoxvirus*	crocodiles
	*Leporipoxvirus*	lagomorphs (rabbits and hares) and squirrels
	*Macropoxvirus*	kangaroo
	*Molluscipoxvirus*	humans, chimpanzees, and donkeys
	*Mustelpoxvirus*	sea otters
	*Orthopoxvirus*	wide range of mammals, including primates and rodents
	*Oryzopoxvirus*	sentinel mouse
	*Parapoxvirus*	cows, goats, and gray seals
	*Pteropoxvirus*	Australian little red flying fox
	*Salmonpoxvirus*	Atlantic salmon
	*Sciuripoxvirus*	red squirrels
	*Suipoxvirus*	swine
	*Vespertilionpoxvirus*	North American brown bat
	*Yatapoxvirus*	primates (monkeys and baboons)
*Entomopoxvirinae*	*Alphaentomopoxvirus*	insects from the order Coleoptera (beetles)
	*Betaentomopoxvirus*	insects from the order Lepidoptera (butterflies and moths)
	*Deltaentomopoxvirus*	insects from the order Orthoptera (North American migratory grasshopper)
	*Gammaentomopoxvirus*	insects from the order Diptera
